# Efficient and precise genomic deletion in rice using enhanced prime editing

**DOI:** 10.1007/s42994-024-00153-9

**Published:** 2024-04-29

**Authors:** Mengyuan Liu, Xiang Zhang, Wen Xu, Guiting Kang, Ya Liu, Xinxiang Liu, Wen Ren, Jiuran Zhao, Jinxiao Yang

**Affiliations:** Beijing Key Laboratory of Maize DNA Fingerprinting and Molecular Breeding, Beijing Academy of Agriculture and Forestry Sciences, Beijing, 100097 China

**Keywords:** Prime editing, Precise genomic deletion, CRISPR, Cas9

## Abstract

Efficient and precise genomic deletion shows promise for investigating the function of proteins in plant research and enhancing agricultural traits. In this study, we tested the PRIME-Del (PDel) strategy using a pair of prime editing guide RNAs (pegRNAs) that targeted opposite DNA strands and achieved an average deletion efficiency of 55.8% for 60 bp fragment deletions at six endogenous targets. Moreover, as high as 84.2% precise deletion efficiency was obtained for a 2000 bp deletion at the *OsGS1* site in transgenic rice plants. To add the bases that were unintentionally deleted between the two nicking sequences, we used the PDel/Syn strategy, which introduced multiple synonymous base mutations in the region that had to be patched in the RT template. The PDel/Syn strategy achieved an average of 58.1% deletion efficiency at six endogenous targets, which was higher than the PDel strategy. The strategies presented in this study contribute to achieving more accurate and flexible deletions in transgenic rice plants.

**Supplementary Information:**

The online version contains supplementary material available at 10.1007/s42994-024-00153-9.

Dear Editor,

The ability to precisely delete specific genomic DNA sequences is important for plant functional genomics. Several methods have been reported for genomic deletions using CRISPR–Cas9-based technologies. Paired Cas9 nuclease that generates DNA double-strand breaks (DSBs) can mediate genomic deletion with unintended mutations (Hsu et al. 2014). The newly developed prime editors (PEs) could be used to program deletions ranging from 5 to 80 bp with high efficiency and modest precision in human cells (Anzalone et al. 2019; Nelson et al. 2022). A pair of prime editing guide RNAs (pegRNAs) targeting the opposite DNA strands achieved precise sequence deletion between two nicks at human genomic sites (Anzalone et al. 2022; Choi et al. 2022). In plants, precise deletions were obtained in rice protoplasts using an engineered plant PE with low efficiency (Zong et al. 2022). In this study, we tested three strategies and achieved as high as 88.0% precise deletion efficiency in transgenic rice plants.

It was reported that both Cas9max nickase (Cas9maxn) and engineered guide RNA (epegRNA) with enhanced 3′ extended tevopreQ1 motif can improve the PE efficiencies in plants (Li et al. 2022; Xu et al. 2022); therefore, we applied them in our PE-P3 system. The classic PE3 strategy was first used to test the deletion efficiency (Fig. 1A and C). We designed pairs of single guide RNAs (sgRNAs) specifying 25 to 60 bp deletions within the promoter or coding region at six loci using the PE3 strategy. Vectors were introduced into rice calli by *Agrobacterium*-mediated transformation to obtain transgenic plants. Genomic DNA was harvested, and primers which specifically recognize the correct deletions were designed to preliminarily screen samples using PCR. Then the regions covering the deletion were amplified and sent for next-generation sequencing (NGS) to further confirm the precise deletions (Supplementary Table 1). NGS revealed that the precise deletion efficiency with the PE3 strategy ranged from 8.3 to 67.5% at the six tested sites, with an average efficiency of 35.6%. Moreover, approximately 2.5 to 25% of undesired indels were generated, with an average efficiency of 13.1% (Fig. 1E and Supplementary Table 2). Among the precise deletion samples, 70.4% and 7.7% of homozygous edits were obtained at the *OsWaxy-P1* and *OsD18* targets, respectively, but most of the edits were heterozygous and chimeric at other targets (Fig. 1F and Supplementary Table 2). This result indicated that the PE3 strategy was able to achieve precise deletion in plants but also included unintended indels and chimeric cells.

**Fig. 1:**
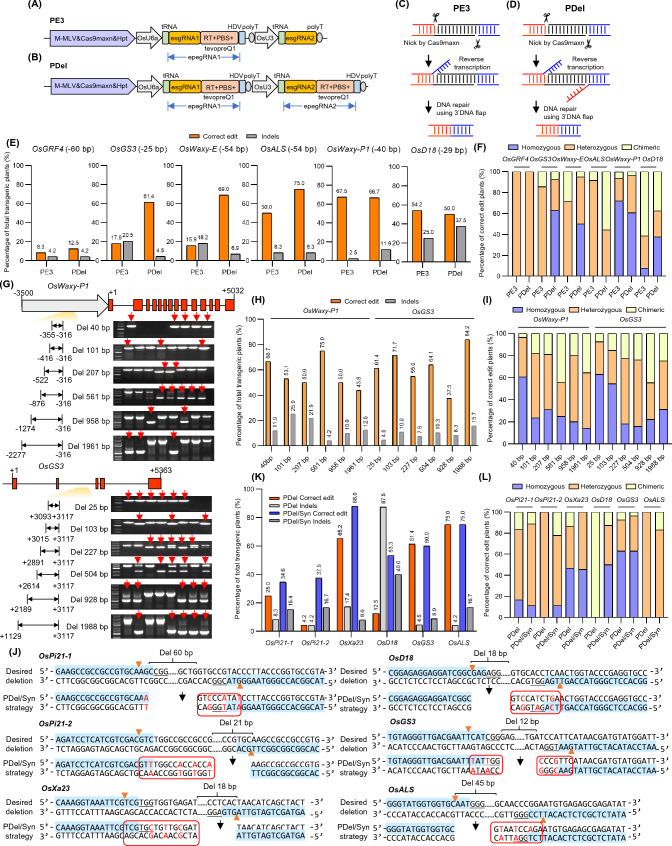
Precise deletions in transgenic rice plants using different strategies. **A** and **B** Schematic view of the vectors using the PE3 strategy (**A**) and the PDel strategy (**B**). **C** and **D**. Schematic model of the deletion process using the PE3 strategy (**C**) and the PDel strategy (**D**). **E** Deletion efficiencies at six genomic loci using different strategies. **F** Proportions of homozygous, heterozygous, and chimeric mutations among the precise deletion plants using PE3 and PDel strategies for short fragment deletions. **G** Representative deletions of different lengths programmed with the PDel strategy at the *OsWaxy-P1* and *OsGS3* sites. A tris–acetate-EDTA (TAE) agarose gel of PCR amplicons for preliminary detection of precise deletions is shown. Samples with red arrows indicated potential precise deletion events. For relative smaller deletions (≤ 500 bp), the marker sizes were 2000, 1500, 1000, 750, 500, 250, and 100 bp. For relatively larger deletions (> 500 bp), the marker sizes were 5000, 3000, 2000, 1500, 1000, 750, 500, 250, and 100 bp. **H** Deletion efficiencies of different lengths at the *OsWaxy-P1* and *OsGS3* sites. **I** Proportions of homozygous, heterozygous, and chimeric mutations among the precise deletion plants using the PDel strategy for long fragment deletion. **J** Design of the PDel/Syn strategy. Red letters indicated the induced synonymous base mutations. Yellow triangles indicated the cut sites of Cas9. Red borders indicated the region of location in the pegRNA pair. **K** Comparison of the deletion efficiencies using PDel and PDel/Syn strategies. **L** Proportions of homozygous, heterozygous, and chimeric mutations among the precise deletion plants using the PDel/Syn strategy for precise deletion

It was reported that the PRIME-Del (herein referred to as PDel) strategy, which includes a pair of pegRNAs, induced efficient sequence deletion with much higher precision than the PE3 strategy in human cells (Choi et al. 2022). Therefore, we tested the PDel strategy using a dual pegRNAs at both the forward and reverse DNA strands in rice (Fig. 1B and D). After preliminary PCR screening, the NGS results of transgenic plants showed that 61.4% and 69% of desired deletions were obtained at the *OsGS3* and *OsWaxy-E* targets, respectively; these values were 3.4- and 4.3-fold higher than those obtained using the PE3 strategy (Fig. 1E). Furthermore, 12.5% (VS 8.3%) and 75% (VS 50%) of precise deletions were generated at *OsGRF4* and *OsALS* targets; these values were 1.5-fold higher than those obtained using the PE3 strategy (Fig. 1E). For the *OsWaxy-P1* and *OsD18* targets, equal percentages of desired deletions were produced by both strategies (Fig. 1E). The average precise editing efficiency of the PDel strategy was 55.8% at the six edited targets, which was higher than the PE3 strategy. Unintended indels also occurred using the PDel strategy with an average frequency of 12.2%, which was equal to that produced by the PE3 strategy (Fig. 1E and Supplementary Table 2). Moreover, among the precise deletions in seedlings, 63% and 50% of homozygous deletions were obtained at *OsGS3* and *OsWaxy-E* targets, respectively, using the PDel strategy, whereas no homozygous deletions were detected using the PE3 strategy at the same targets (Fig. 1F). Although the proportion of intended deletions at *OsD18* was similar between the two strategies, the PDel strategy generated more homozygous edits than the PE3 strategy (37.5% Vs 7.7%) (Fig. 1E and F). Collectively, the increased frequency of intended deletions and the higher proportion of homozygous edits indicated that the PDel strategy has great potential to induce precise deletions in rice.

Encouraged by the above result, we then expanded the range of deletion lengths using the PDel strategy. DNA fragment deletions that ranged from 100 to 2000 bp were designed at the *OsWaxy-P1* and *OsGS3* loci (Fig. 1G). Deletions were first identified by electrophoresis based on the product differences between the wild-type and the deleted fragments before NGS (Fig. 1G). Then, NGS revealed that 37.5% to 84.2% of correct deletions of different lengths were obtained at both *OsWaxy-P1* and *OsGS3*. In particular, 43.8% and 84.2% of ~ 2000 bp deletions were observed at the *OsWaxy-P1* and *OsGS3* loci, respectively (Fig. 1H and Supplementary Table 3). This result indicated that the deletion frequency was not affected by deletion lengths from 50 to 2000 bp. Among the mutants, 14.3% to 63% of homozygous deletions were detected for all the different deletions (Fig. 1I). The percentage of homozygous deletions steeply decreased from 60 to 23.5% when the deletion length increased from 40 to 101 bp at the *OsWaxy-P1* site (Fig. 1I). The same phenomenon was observed at the *OsGS3* site when the deletion length increased from 103 to 227 bp (Fig. 1I). These results show that the PDel strategy is capable of obtaining high efficiency of precise deletions that range from 50 to 2000 bp of length in transgenic rice plants.

Although highly precise deletion can be achieved using the PDel strategy, some regions could not be fully deleted because of the limitation of the PAM location in the pegRNA pair. Moreover, it was reported that if the RT template contained a homologous sequence to the target region, the deletion or insertion efficiency would be decreased compared with those without a homologous sequence (Wang et al. 2022). Therefore, we induced multiple synonymous base mutations in the region that had to be patched in the RT template, which was referred to as the PDel/Syn strategy (Fig. 1J). Six loci was tested (*OsPi21*, *OsPi21-2*, *OsXa23*, *OsD18*, *OsGS3*, and *OsALS*), and the PDel strategy was used as the control. After preliminarily PCR screening used specific primers and NGS analysis of the transgenic plants, the PDel/Syn strategy greatly improved the precise deletion efficiency at *OsPi21-1* (25% up to 34.6%), *OsPi21-2* (4.2% up to 37.5%), *OsXa23* (65.2% up to 88%), and *OsD18* (12.5% up to 53.3%) sites compared with the PDel strategy. Similar precise deletion efficiencies were found at *OsGS3* and *OsALS* sites with both PDel/Syn and PDel strategies (Fig. 1K and Supplementary Table 4). The average precise deletion efficiency improved from 40.6 to 58.1% using the PDel strategy compared with the PDel/Syn strategy (Fig. 1K and Supplementary Table 4). Additionally, the proportions of homozygous deletions obtained at *OsPi21-2* and *OsD18* were 11.1% and 50%, respectively, by employing the PDel/Syn strategy, but homozygous deletions were undetected at these sites using the PDel strategy (Fig. 1L). The above results indicated that the PDel/Syn strategy has the potential advantage of achieving more precise deletion events as desired, with higher proportions of homozygous mutations.

In the present study, we tested three strategies (PE3, PDel, and PDel/Syn) for precise deletion of the target segment, and all three methods successfully achieved the desired deletion. Improved efficiencies with increased homozygous mutations of precise deletions were obtained using PDel strategy in transgenic rice plants. The deletion segment length ranged from 50 to 2000 bp, which may have the potential to be longer using the PDel strategy. Moreover, the PDel/Syn strategy induced synonymous amino acids in the genome to avoid unnecessary side effects of deletion and facilitated patching of useful sequences that were inadvertently deleted during the PDel strategy, which can help achieve more accurate and more flexible deletions. In future research, different Cas9 variants with expanded PAMs and smaller sizes should be tested in PDel and PDel/Syn systems. Furthermore, this expanded capability may provide a new tool for the precise deletion of promoters or protein functional domains of the target sites, which will enable the study of the physiological function and signal transduction processes across a wide range of plant research and agricultural applications.

## Materials and methods

### Plasmid construction

The plasmids were constructed as the previously reported PE-P3 vector (Xu et al. 2022) with the following modifications. The epegRNA contained a 20 bp target, enhanced sgRNA (esgRNA) (Chen et al. 2013), RT template, PBS sequence, linker, and tevopreQ1. The esgRNA and tevopreQ1 + HDV element were individually synthesized and cloned into the pUC-57 vector to be the amplifying template. Target, RTT PBS sequences, and linker were synthesized within the forward and reverse primers. For the PE3 strategy, OsU6a + tRNA, an esgRNA fragment with a 20 bp target at the 5′ terminus and RTT PBS at the 3′ terminus, and a tevopreQ1 + HDV fragment with linker at the 5′ terminus were treated with NEBuilder HiFi DNA Assembly Master Mix (New England Biolabs) to generate the epegRNA cassette. The epegRNA cassette was ligated to the PE-P3 vector to generate the final plasmid. For the PDel strategy, two epegRNA cassettes were produced as described above to generate the final plasmid.

### Agrobacterium transformation of rice callus cells

The rice cultivar Nipponbare (*Oryza sativa* L. *japonica* cv. Nipponbare) was used in this study. All binary vectors were introduced into *Agrobacterium tumefaciens* strain EHA105 using a freeze–thaw method. Rice embryogenic calli were then infected by *Agrobacterium*, as previously described (Hiei and Komari 2008). The incubated and recovered calli were selected on medium containing 50 mg/L hygromycin for 4 weeks to obtain resistant calli. Vigorously growing calli were then transferred to regeneration medium to generate green plants.

### Plant DNA extraction and preliminary detection of deleted samples

Generally, genomic DNA was extracted from transgenic plants using the DNA Quick Plant System (Tiangen Biotech, Beijing, China). Different primer pairs were designed for the short (< 100 bp) and long (≥ 100 bp) fragment deletions. For the preliminary detection of short fragment deletions, unique sequences were designed that exactly matched the precise deletion region, and the other primer was located in the undeleted genomic region; therefore, they could only amplify the samples with precise deletion. For sequence confirmation (including heterozygous and homozygous) of short deletions, primers located upstream and downstream of the deletion region were designed, and the PCR product was sent for NGS. To preliminarily detect long deletions, primers located upstream and downstream of the deletion region were designed that amplify different lengths of PCR products that can be separated by 2.0% agarose gel. For heterozygous and homozygous confirmation of longer deletions, one unique primer for the deleted region and one primer for the undeleted region were designed to eliminate amplification preference from the PCR method.

### Amplicon deep sequencing and data analysis

The target region was amplified from all DNA with primers in the first round of PCR (Supplementary Table 1). In the next round of PCR, both forward and reverse barcodes were added to the ends of the PCR products using barcode primers. The PCR products were sent for NGS (Tsingke Biological Technology, Beijing, China) using the MiSeq platform. For relatively smaller deletions (generally less than 60 bp), analysis of prime-editing mutations were performed using GATK software and CRISPResso2 (Clement et al. 2019) to call indels. For larger deletion (hundreds of base-pair), calling indels/SNP and de novo assembly were both used for each NGS data. Mutations reads that were present in fewer than 5% of transgenic plants were filtered during data analysis. Percentage of total transgenic plants was calculated as: number of plants with precise or imprecise edits divided by the number of NGS samples. Percentage of correct edit plants was calculated as the number of homozygous, heterozygous, or chimeric mutations divided by the number of plants with correct edits. GraphPad Prism v.8 (GraphPad Software, San Diego, California USA, www.graphpad.com) was used to analyze all the data. The sequencing depth of each sample was about 200 × . Examples of NGS data for PE3, PDel and PDel/Syn strategies with scripts for analyzing mutations are available at https://github.com/zhangxiang36/PDel.

### Supplementary Information

Below is the link to the electronic supplementary material.

Supplementary file1 (XLSX 21 KB)

Supplementary file2 (PDF 161 KB)

## Data Availability

All data supporting the findings of this study are available within the paper and supplementary information.
