# Supplementary material for: Efficient and precise genomic deletion in rice using enhanced prime editing
Source: aBIOTECH. 2024 Apr 29;5(2):214–8. doi: 10.1007/s42994-024-00153-9 (PMC11224055; doi:10.1007/s42994-024-00153-9)
Supplement: Supplementary file 2 — Supplementary file2 (PDF 161 KB) [file 42994_2024_153_MOESM2_ESM.pdf]

(a) **PE3**

OsWaxy-P1 desired: -40 bp

```
       1631      1641      1651      1661      1671      1681
GCAACGTACGCCAAGCCGAAACGGCAGGCAGCATCGCGCACGCACGCACACACAGGCCACAGCACACGCG
......................****************************************........ 82
............T.........****************************************........ 1
......C...............****************************************........ 1
```

(b) **PDel**

OsGS3 desired: -25 bp

```
 5101      5111      5121      5131      5141      5151
AATTTTTTTGTAGGGTTGACGAATTCATCGGAAGAACTCCTGATCCATTCATAACGATGTATGGATTTTC
.........................*************************.................... 383
..*......................*************************.................... 1
.........................*************************...............A.... 1
.........................*************************..T................. 1
...................T.....*************************.................... 1
```

(c) **PDel/Syn**

OsD18 desired: -18 bp

```
        2691      2701      2711      2721      2731      2741
AGTCGAGGCGGAGAGGAGGATCGGCGAGAGGATGACGGCGACGGTGCACCTCAACTGGTACCCGAGGTGC
.........................******************..C..T..G.................. 170
.........................******************..C..T..G........T......... 1
...................................................................... 1
.............................................C..T..G.................. 1
...........G.............******************..C..T..G.................. 1
```

Supplementary Figure 1 Sequencing data of the precise deletions of PE3, PDel and PDel/Syn. "*" represents deletion, "." means the same nucleotide with wild type. Red letters in (c and d) represent positions of multiple synonymous base mutations. Reads fully covering the target region are extracted and counted, and numbers of every kind of reads are shown on the right of the sequence of reads.

(a) **PE3**

OsWaxy-E desired: -54 bp

```
      2261      2271      2281      2291      2301      2311
GACCTGACACTGGAGTTGATTACAAAGACAACCAGATGCGTTTCAGCCTTCTTTGCCAGGTCAGTGATTA
.......******************************************************......... 73
......*****..*********G......********************************......... 59
...................................................................... 5
..T...*****..*********G......********************************......... 1
```

(b) **PDel**

OsGS3 desired: -25 bp

```
 5101       5111      5121      5131      5141      5151
AA*TTTTTTTGTAGGGTTGACGAATTCATCGGAAGAACTCCTGATCCATTCATAACGATGTATGGATTTTC
..*.......................*************************.................... 266
..*..............***************............A.G.A.T.................... 60
..*..............***************............A.G.A.T................G... 1
..*.......................*************************.............A...... 1
..*.......................*************************.......A............ 1
..*.......................*************************....T............... 1
..*.................................................................... 1
..*................G......*************************.................... 1
..*..........T............*************************.................... 1
..*.........T.............*************************.................... 1
..T.......................*************************.................... 1
```

(c) **PDel/Syn**

OsD18 desired: -18 bp

```
      2691      2701      2711       2721      2731      2741
AGTCGAGGCGGAGAGGAGGATCGGCGAGAGGATGA*CGGCGACGGTGCACCTCAACTGGTACCCGAGGTG
.........................*******************..C..T..G................. 160
................................G..T....****..C..T..G................. 34
.........................*******************..C..T..G...............C. 1
........................T.......G..T....****..C..T..G................. 1
......................T..*******************..C..T..G................. 1
.................A.......*******************..C..T..G................. 1
```

Supplementary Figure 2 Sequencing data of the byproducts edits of PE3, PDel and PDel/Syn. "*" represents deletion, "." means the same nucleotide with wild type. Red letters in (c and d) represent positions of multiple synonymous base mutations. Reads fully covering the target region are extracted and counted, and numbers of every kind of reads are shown on the right of the sequence of reads.
